# Achieving a Balanced Knee in Robotic TKA

**DOI:** 10.3390/s21020535

**Published:** 2021-01-13

**Authors:** Alexander C Gordon, Michael A Conditt, Matthias A Verstraete

**Affiliations:** 1 Illinois Bone and Joint Institute, Des Plaines, IL 60016, USA; agordon@ibji.com; 2 OrthoSensor Inc., Dania Beach, FL 33004, USA; michael.conditt@orthosensor.com

**Keywords:** balancing, robotic total knee arthroplasty, alignment

## Abstract

Total knee arthroplasty (TKA) surgery with manual instruments provides a quantitatively balanced knee in approximately 50% of cases. This study examined the effect of combining robotics technology with real-time intra-operative sensor feedback on the number of quantitatively balanced cases in a consecutive series of 200 robotic-assisted primary TKAs. The robotics platform was used to plan the implant component position using correctable poses in extension and a manual, centrally pivoting the balancer in flexion, prior to committing to the femoral cuts. During the initial trialing, the quantitative state of balance was assessed using an instrumented tibial tray that measured the intra-articular loads in the medial and lateral compartments. These sensor readings informed a number of surgical corrections, including bone recuts, soft-tissue corrections, and cement adjustments. During initial trialing, a quantitatively balanced knee was achieved in only 65% of cases. After performing the relevant soft-tissue corrections, bone recuts, and cement adjustments, 87% of cases ended balanced through the range of motion. Meanwhile, this resulted in a wide range of coronal alignment conditions, ranging from 6° valgus to 9° varus. It is therefore concluded that gaps derived from robotics navigation are not indicative for a quantitatively balanced knee, which was only consistently achieved when combining the robotics platform with real-time feedback from intra-operative load sensors.

## 1. Introduction

**Figure 1:**
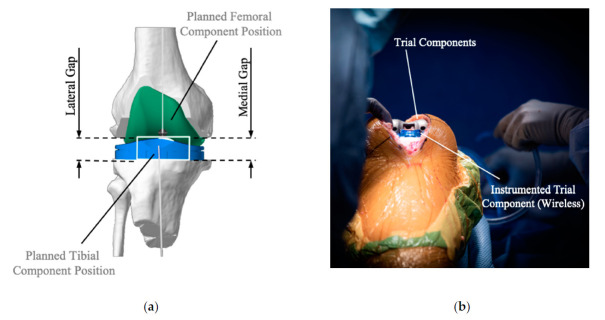
Virtual component planning during surgery allowing to assess the resulting space between the resected bones, represented by the (**a**) medial and lateral gaps and (**b**) use of intra-operative sensor technology to assess the tibiofemoral loads with the trial components in place.

Total knee arthroplasty has been a successful intervention to relieve pain and restore function in patients with end stage gonarthrosis. The principles and practices of this operation have evolved with experience, but some fundamentals remain consistent. The concepts of joint alignment and ligament balance are important factors in the success of total knee arthroplasty (TKA), but some controversy still exists concerning the optimal alignment and balance targets for a given patient [1,2,3]. Intra-operative limb alignment has traditionally been assessed using manual instruments, with some degree of inaccuracy. Computer assisted navigation systems (CAS), introduced in the 2000s, offered promise of improved alignment and outcomes. Studies have indicated that the use of CAS can decrease alignment outliers but may not have a large effect on outcomes. Within the available CAS systems, ligament balance data was offered to a surgeon by way of numerical gap measurements. Numerical gap measurements show the surgeon before any cuts have been made and the virtual distances between the proposed cut surfaces in the medial and lateral compartments of the knee in flexion and extension (Figure 1a for example of extension gaps). The surgeon can then adjust the placement plan of the components to provide a desired gap for each of the four measurements (extension and flexion in combination with respectively medial and lateral side of the knee) [4]. In contrast, an intra-operative kinetic balancing system has been introduced to be used with a variety of total knee systems. This device offers surgeons a quantitative assessment of load across the joint throughout an arc of motion and is used after the cuts have been made and with the trial components in place [5]. The load measurement device is located in the tibial trial insert and provides the loads in each compartment throughout the range of motion (Figure 1b).

Recent studies have indicated that patient reported outcomes are improved if the knee is quantitatively balanced as compared to unbalanced knees [6,7,8]. Robotics has entered the field in total knee arthroplasty. Various robotic systems have been available to orthopedic surgeons, dating back to the ACROBOT system in the late 1980s up to both image-based and image-less systems in the market today [9]. One particular system, using a haptic-guided robotic arm, offers the surgeon 3D planning with a preoperative computed tomography (CT) scan, with intra-operative modifications based on the surgeon’s dynamic assessment. The robotic arm is used as the execution tool for the plan rather than a traditional saw and cutting block. The registration process and gap information given to the surgeon is similar to the information previously provided by CAS.

This paper evaluates the success of achieving a quantitatively balanced knee using a robotics platform and the potential advantages of complementing the robotics platform with sensor feedback to achieve a quantitatively balanced knee. Therefore, the primary research question is how robotically derived flexion and extension gaps correlate with intra-articular loads measured by the sensor system during trialing. Additionally, this research aims to understand what intra-operative maneuvers are subsequently indicated to achieve a quantitatively balanced knee when combining these technologies and how this is reflected in the final coronal alignment and the balance through the range of motion. 

## 2. Materials and Methods

### 2.1. Surgery

The study cohort consists of 200 patients who underwent total knee arthroplasty using a haptic-guided robotic system (Mako, Stryker Corp, Mahwah, NJ, USA) and an intra-operative load sensor (VERASENSE, OrthoSensor Inc, Dania Beach, FL, USA). All cases were performed by a single surgeon (A.G.) using a cruciate retaining implant system (Triathlon, Stryker Corp, Mahwah, NJ, USA). The surgeon’s target for completion of each case was quantitative balance throughout the range of motion, represented by an absolute load in both compartments between 10 and 35 lbf with a medio-lateral differential not exceeding 15 lbf [10,11]. Coronal limb alignment deviations from a neutral mechanical axis were tolerated and not corrected if the knee was in a balanced state. The surgical technique involved a pre-operative CT scan and planning based on bone anatomy. Coronal alignment, sagittal alignment (contracture), and gaps in flexion and extension were assessed after registration, with modifications made to the distal femur and proximal tibia plan to optimize the extension space. Therefore, the surgeon assessed the correctable coronal alignment in extension first. The surgeon set an arbitrary boundary of 2° deviation from neutral mechanical alignment on the individual extension gap bone cuts. Following the tibial cut, the flexion gap was constructed using a manual central pivot tensioner with the knee at 90° flexion, with modifications made to the femoral rotation, anteroposterior (AP) position, and size on the plan to optimize the flexion space. The tibial slope was constrained to three degrees posterior slope. After bone resection to this plan, a preliminary trialing was performed with the intra-operative load sensor showing the compartmental loads. If severely asymmetric loads were encountered, bone modifications were performed. A second trialing with the load sensors was subsequently performed, and if an unbalanced state was still present, soft tissue adjustments were made. Small amounts of asymmetry were addressed with cement adjustments at final implantation. 

Surgical corrections of an imbalanced knee were subdivided into three groups: bone recuts, soft tissue adjustments, and cement adjustments. The choice of adjustment was based on the state of balance at a particular time in the operation. When a large load imbalance was measured (>70 lbf loads in either or both compartments) at the preliminary trialing after executing the initial planned cuts, with a “floating” tibia tray, bone recuts were performed. The location of bone cuts was guided by the flexion angles and compartments where excessive loads were observed. When observing high loads through the range of motion for a given compartment, a tibial recut was preferred. For small overload conditions, these involved freehand skim-cuts, whereas robotic cuts were considered after adjusting the plan. However, if the high loads were isolated to either the flexion or extension space, a femoral recut was performed to proximalize or rotate the femoral component using the robotics platform. Once the tibia had been prepared and a tibial trial tray was fixed in place, soft tissue adjustments were performed if load imbalance still exceeded 15 lbf. In cases of high medial loads in extension, the deep medial collateral ligament and posteromedial capsule were released from the tibial joint line allowing for complete osteophyte removal. The posterior cruciate ligament (PCL) was released sharply from its femoral attachment in a titrated manner in cases with high medial loads in flexion with a posterior medial centroid of load. Pie crusting of the medial collateral ligament (MCL) was performed using an 18-gauge needle when high medial loads were noted in extension after the previous joint line release had been performed. Lateral soft tissue releases were less common but utilized to correct high lateral loads. In extension, the posterolateral capsule was targeted with a joint line release, and in flexion, the popliteus was released from its femoral attachment. A lateral retinacular release was only utilized to correct patella tracking, not to correct inter-compartmental loads. In some cases, during cementation and implantation, asymmetrical loads were still encountered. At those times, differential impaction at the time of component cementation for the medial/lateral tibia or distal femur was used as a method to correct imbalances at the final stage of the operation. 

### 2.2. Data and Statistics

During surgery, intra-operative data was collected from both the robotics and sensor platform. This data involved coronal and sagittal overall limb alignment prior to the initial bone resections. The planned component positions, as well as bone resections, were also recorded. During subsequent trialing, the intra-articular loads were recorded at 10°, 45°, and 90° flexion, while also recording the coronal and sagittal alignment. Following the surgical corrections, the alignment and load parameters are assessed and recorded again at the given flexion angles. 

Following the intra-operative data collection, the data was collected in a database and subsequently analyzed and visualized using Python version 3.6. Statistical analysis has been performed using Python’s SciPy package (Mann–Whitney U-test).

## 3. Results

### 3.1. Patient Demographics

In our population of 200 consecutive primary total knee patients, 71.9% represented female patients. Overall, our population had a mean age of 70.1 years, with an average body mass index (BMI) of 30.4 kg/m^2^ with a standard deviation of 5.4 kg/m^2^. The pre-operative coronal alignment ranged between 24° valgus and 25° varus, with a mean pre-operative varus alignment of 4.7° (Table 1).

### 3.2. Planned Gaps

**Figure 2:**
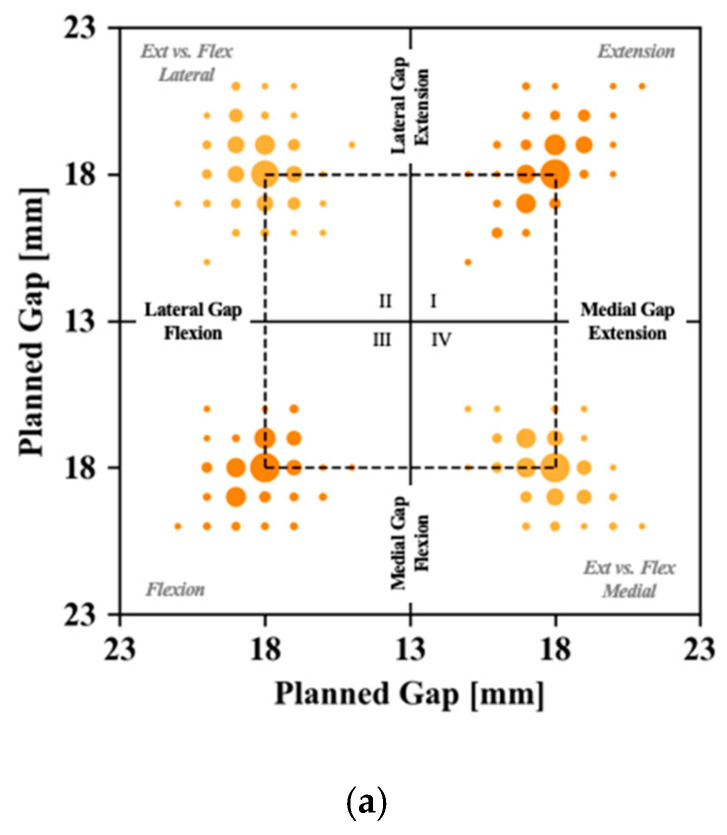

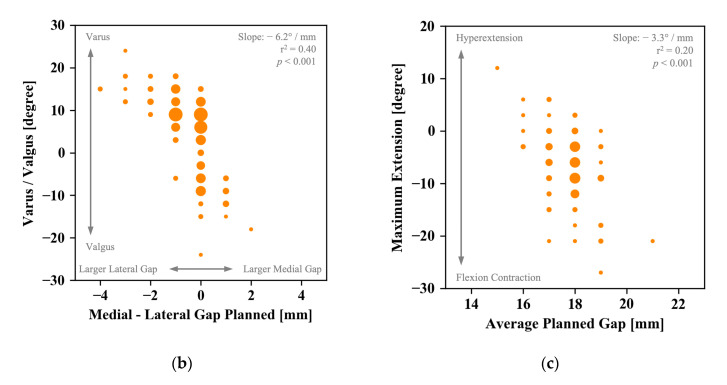
(**a**) Distribution of planned gaps medially and laterally in flexion and extension. The dotted line shows gaps planned at 18 mm throughout. The first quadrant (I) compares the medial versus lateral gap in extension. The third quadrant (III) compares the medial versus lateral gaps in flexion. The second (II) and fourth (IV) quadrants compare the lateral respectively medial gaps in flexion versus extension. (**b**) Correlation between coronal alignment in extension and mediolateral difference in planned gaps in extension and (**c**) correlation between maximum sagittal alignment in extension and average mediolateral planned gaps in extension. Throughout this figure, the diameter of the dots is representative for the number of cases presenting with the respective conditions.

Prior to performing the bone cuts, the cutting plan was adjusted based on the correctable alignment in extension and the read-out of a manual, not the instrumented tensor in flexion. This resulted in a wide distribution of planned gaps between the resected bones, not necessarily reflecting the symmetrical 18 mm thickness of the implant components that were introduced (Figure 2a shows a dotted line representing 18 mm gaps). Deviations from the theoretical 18 mm gap were guided by the coronal alignment, as shown in Figure 2b, where an increased pre-operative varus alignment significantly correlated to a relatively larger lateral than medial gap (*p* < 0.001). Similarly, a lack of (or excessive) terminal extension also affected the planned gaps (Figure 2c). More specifically, a lack of terminal extension was significantly correlated to a larger average gap between the medial and lateral compartment (*p* < 0.001).

### 3.3. Sensor Feedback and Surgical Corrections 

**Figure 3:**
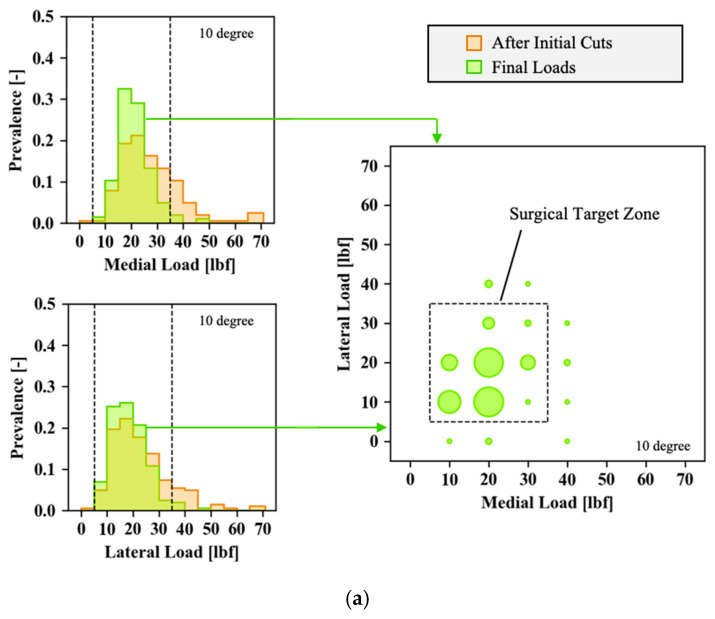

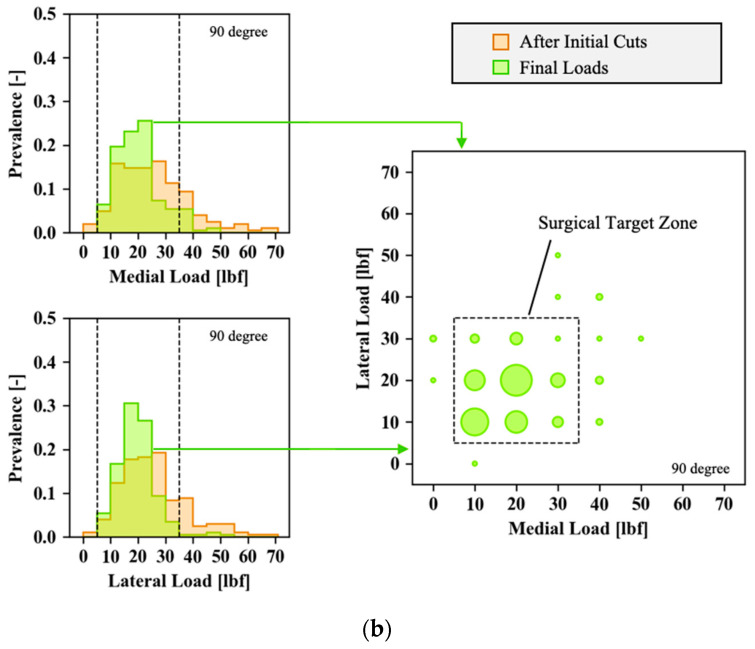
Correlation between mediolateral loads in (**a**) 10° and (**b**) 90° knee flexion. The left-hand side shows the relative medial (upper) and lateral (lower) load distributions before (red) and after (green) the surgical corrections have been completed, bucketed in 5-lbf intervals. Building on the final loads, after the surgical corrections have been completed, the correlation between the medial (*x*-axis) and lateral (*y*-axis) loads is shown on the right-hand side. The diameter of the green circles is indicative for the number of cases presenting with a given load distribution.

Following the initial cuts, and with the trial components in place, the intra-articular loads are widely scattered, as shown by the distribution in red in Figure 3. Both condyles with 0 lbf of intra-articular load and loads in excess of 70 lbf are observed during initial trialing.

Looking at both medial and lateral loads at 10° and 90° flexion, 80.4% of cases show loads between 5 and 35 lbf per compartment during the initial trialing, with an overall average (standard deviation) load per compartment of 23.0 (13.0) lbf (details per compartment shown in Table 2). The subsequent balancing steps significantly reduce the joint load to an average (standard deviation) of 17.4 (7.1) lbf, with 96.1% of loads ranging between 5 and 35 lbf. The final conditions also show that the medial loads trend towards higher forces relative to the lateral loads, particularly in extension.

**Figure 4:**
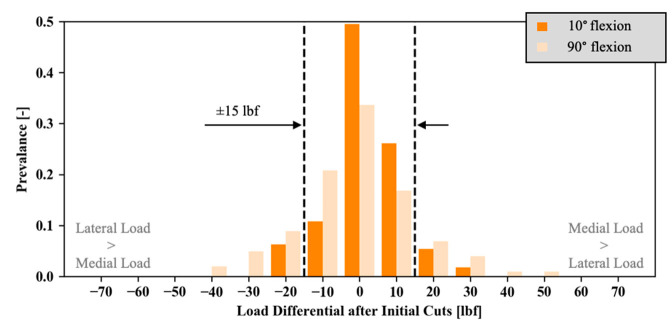
Distribution of intra-articular load differentials during initial trialing for those cases planned with equal, symmetrical gaps in extension (darker tone) and 90° flexion (lighter tone).

Focusing on the load difference between both compartments, only cases with equal planned gaps are considered to avoid a potential bias by cases intentionally planned with larger medial or lateral gaps. The load difference during initial trialing for these cases, at 10° and 90° flexion, is shown in Figure 4. At 10° flexion, 86.1% of the selected cases have an absolute mediolateral load differential less than 15 lbf. At 90° flexion, 71.3% of these cases have an absolute mediolateral load differential less than 15 lbf. In the latter cases, load differentials up to 50 lbf are encountered during initial trialing. 

**Figure 5:**
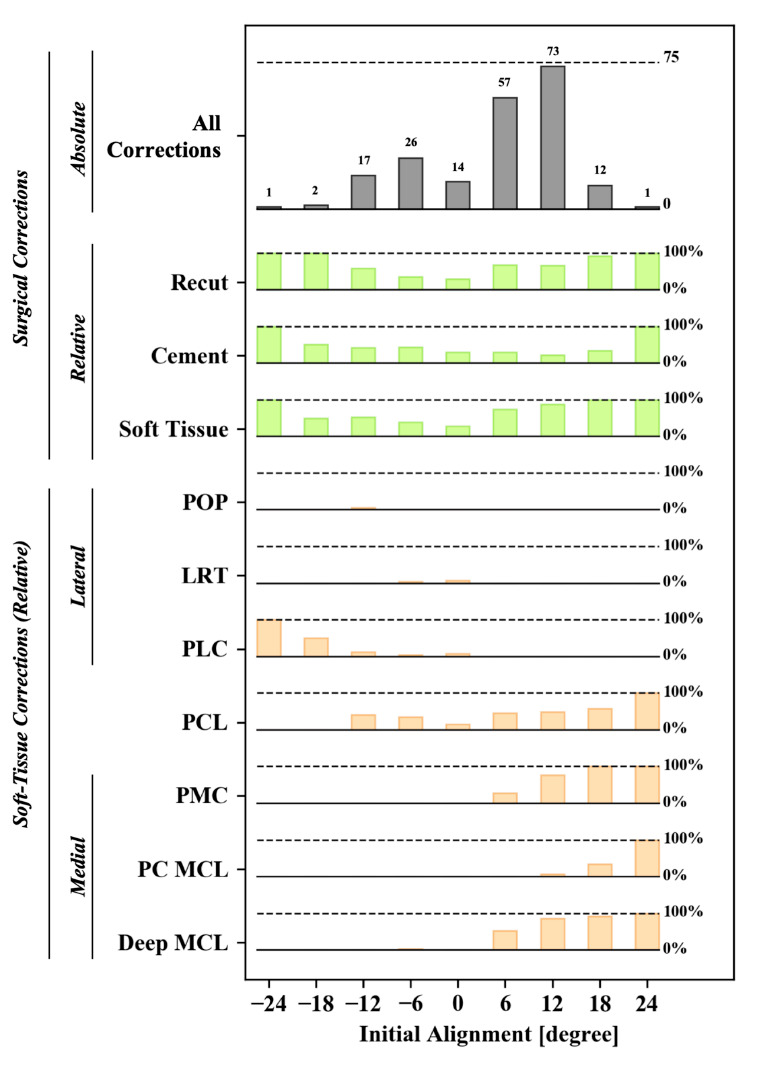
Distribution of absolute (top bar chart) and relative prevalence of surgical corrections to balance a total knee following the initial cuts as a function of the initial coronal alignment. Overall, three different types of surgical corrections can be selected, whose relative prevalence is shown in green bars for bone recuts, cement adjustments, and soft-tissue adjustments, respectively. The latter, the orange corrections, represent all the different soft tissue corrections documented throughout the study; popliteus release (POP), lateral retinaculum release (LRT), posterior lateral corner release (PLC), posterior cruciate ligament release (PCL), posterior medial corner release (PMC), pie-crusting of the medial collateral ligament (PC MCL), and release of the deep medial collateral ligament (deep MCL).

To address the encountered imbalance, as well as the low or excessive loads in the knee, various surgical corrections are subsequently performed. In Figure 5, an overview is given of the (relative) prevalence for the various surgical corrections as a function of the pre-operative deformation. From this graph, it is clear that soft tissue, bone, and cement adjustments are more likely for knees with a higher degree of pre-operative deformity. Focusing on the soft tissue corrections, lateral corrections (popliteus and posterolateral corner release) are more common in valgus knees, whereas medial releases (posteromedial corner release, pie-crusting of the MCL, and release of the deep MCL) are more common in the pre-operative varus knees. A release of the posterior cruciate ligament is prevalent across all pre-operative alignment conditions. 

### 3.4. Balance through Range of Motion

**Figure 6:**
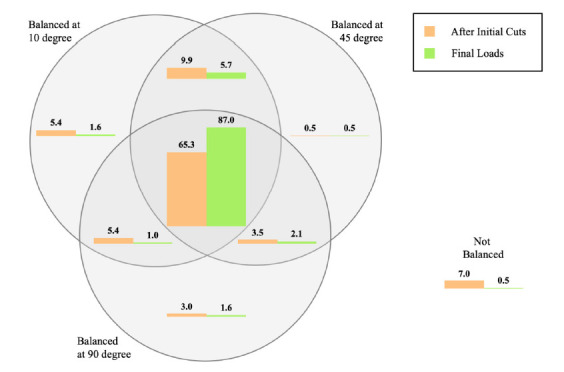
Venn diagram showing the relative distribution (expressed in percentage) of cases achieving quantitative balance at various flexion angles during initial trialing (red) and after surgical corrections were completed (green).

Looking at all cases, 65.3% of cases were quantitatively balanced throughout the range of motion during initial trialing (Figure 6). Meanwhile, 7% of cases were balanced neither at 10°, 45°, or 90° flexion. For those cases that were unbalanced, extension balance was more frequently observed than flexion balance (15.3% respectively 6.5%). 

Following the surgical corrections outlined above, 87% of cases were quantitatively balanced throughout the range of motion and only 0.5% of cases (1 knee) remained unbalanced (Figure 6). Again, for the unbalanced cases, extension balance was more easily achieved than flexion balance. Looking at the various flexion angles, it also clear that mid-flexion (45°) balance was achieved for all but two knees that were balanced at 10° and 90° using this implant system. In other words, 98.8% of knees balanced at 10° and 90° were also balanced at mid-flexion (45° flexion). 

### 3.5. Final Alignment

**Figure 7:**
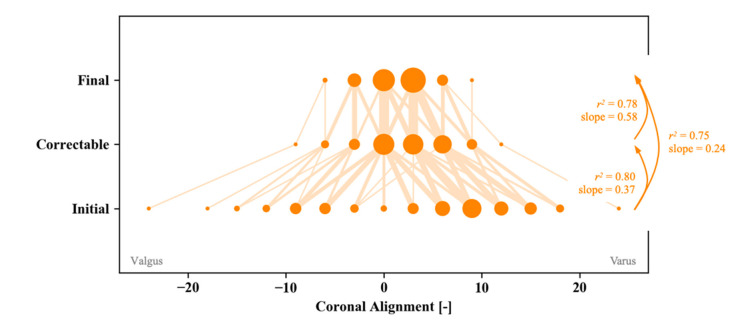
Distribution of initial, correctable, and final coronal alignment in full extension, grouped in 3-degree intervals. The dot size reflects the number of cases in the database that are within the given 3-degree interval for each alignment condition. The lines indicate how the knee alignment correlates with the pre-operative and correctable alignment and the correctable and final alignment. The thickness of the lines thereby reflects the number of knees that follow the shown trajectory.

While aiming to achieve quantitative balance, neutral mechanical alignment was not targeted. This is reflected by the wide distribution of final alignment, as measured using the robotic platform after balancing was completed (Figure 7). As indicated by the (width of the) lines shown in this graph, this figure also illustrates that the initial, pre-operative coronal alignment was generally reduced during surgery. Pre-operatively, coronal alignment between 24° valgus and 25° varus was observed, though the majority of cases presented with a mild (3–15°) varus deformity. During the initial implant planning, the correctable alignment was assessed by manually forcing the leg towards neutral coronal alignment, allowing to read the so-called correctable alignment from the robotic platform. This correctable alignment correlated well with the pre-operative deformity (r^2^ = 0.80) and was a good predictor of the final alignment (r^2^ = 0.78). Nevertheless, no strict 1:1 correlation was observed between the correctable and final alignment, which was linked to the need for additional surgical corrections after the initial bone cuts were completed and the fact that gaps were not necessarily planned symmetrically. The final alignment ranged between 9° varus and valgus, with the majority of cases planned around neutral up to 3° varus coronal alignment.

## 4. Discussion

In this paper, we evaluated the success of achieving a quantitatively balanced knee using the Stryker Mako TKA platform. The results indicate that sensor feedback has the potential to improve the success of achieving a balanced knee through the range of motion; an increase in balanced knees was observed from 67% to 87% after considering sensor feedback, while eliminating cases with load outliers (e.g., >70 lbf per compartment). In addition, our research indicates that knees planned with mediolateral symmetrical flexion and extension gaps can result in a wide range of mediolateral load differentials, suggesting that gaps are a poor surrogate for a quantitatively balanced condition. To achieve a quantitatively balanced knee in this series, the surgeon performed a wide range of soft-tissue corrections in combination with bone recuts and cement adjustments. These were indicated despite the extended range of allowed post-operative coronal alignment conditions and, although primarily focused on sensor readings near extension or 90° flexion, also resulted in a balanced condition in mid-flexion. 

While TKA has proven to be an effective operation for relieving pain and restoring function in patients with end stage degeneration of the knee joint, failures continue to occur [12,13]. Modes of failure, such as aseptic loosening, polyethylene wear, and instability, can often be attributed to the technical performance of the operation [14,15,16,17]. Traditional methods of component alignment and ligament balance are either performed without objective measurement or are subject to measurement errors [18], which contribute to some early and late failures of TKA. Additionally, even with seemingly well-aligned and balanced knees, many patients are dissatisfied with the outcome of their operation. Although patient factors, such as obesity, comorbid conditions, and mental disorders, influence TKA outcomes [19,20], some technical factors may play a role in patient dissatisfaction. Computer assisted orthopedic surgery has been introduced in an effort to reduce outliers and improve outcomes, with imageless navigation systems being most widely utilized and studied. This technology can reduce coronal plane alignment outliers [21,22], though, on the whole, has not been proven to improve patient reported outcomes [23]. Its role in preventing long-term failure is currently being studied, but current guidelines by the American Association of Orthopedic Surgeons do not advocate for its routine use. However, intra-operative load sensors offer surgeons different information than traditional navigation systems. Navigation provides data on a numerical gap, measured medially and laterally in extension and flexion before cuts are made, usually displayed in millimeters. In addition, load sensor data is a function of the soft tissue tension across the knee joint and can be measured throughout the arc of motion with trial components in place, with unit of pounds force. Recent research has demonstrated that when a knee joint is balanced, as defined by the sensor, indicating a medial-lateral difference of less than or equal to 15 lbf, patient reported outcomes are improved compared to unbalanced knees [6,7,8]. Additionally, research has also noted that a surgeon’s subjective assessment of ligament balance is often at odds with sensor readings, with correlation only approximately 50% of the time [8,24,25]. Robotic systems have recently been introduced in an effort to improve outcomes of TKA. In this study, a system that uses a CT-based pre-operative plan and an intra-operative navigation system with a haptic-guided robotic arm was employed. This system gives the surgeon the opportunity to do a dynamic assessment of alignment and stability and make adjustments to the plan to optimize component position. Proponents of this system state that these features allow for better ligament balance and that fewer soft tissue releases are necessary [4]. The purpose of this study was to assess robotically derived and sensor derived data to see if there is a correlation between the gaps noted by the robot and the loads indicated by the sensor. 

The data in this study clearly show that targeting equal pre-cut gaps with the robotic system does not guarantee a balanced knee once those cuts are performed. The primary explanation lies in the manner in which the gap predictions are made. Before any cuts were made, the 3D models of the bones and the associated plan were registered intra-operatively to the actual bone surfaces. The displayed gaps were then the prediction of the distance between the femoral and tibial virtual cut surfaces and were displayed at full extension and 90° flexion. To capture the appropriate “poses” at those two flexion angles to evaluate the gaps, the leg was physically manipulated to stress the collaterals in extension, thus predicting where the limb will be after the cuts and implantation. As it is difficult to apply a varus/valgus stress test in flexion due to the inability to control rotation about the hip, the stresses in flexion have in this study were applied from within the joint with some kind of tensioning device or “spoon” spacers. Neither of these maneuvers quantified the amount of stress being applied externally in extension or tension applied internally in flexion. Thus, the actual joint balance or tension in the soft tissues cannot be quantitatively assessed/predicted in this manner. If the gaps captured during the flexion and extension poses were at least as large as the combined thickness of the metal and plastic implanted, this procedure at least ensures that the implants will fit; however, it does not inform how tight or loose the collaterals are beyond the subjective feel of the surgeon during these maneuvers. After the cuts were made and the trials implanted, the load measuring sensors then evaluated the joint tension/balance with the prescribe cuts, allowing fine tuning of the either the cuts or the soft tissues to achieve a balanced knee, as described in the methods above. It is now clear to see that planning equal gaps that are pre-cut without assessing the tension in the soft tissues (the collaterals in particular) around this “pose” should not be expected to produce a balanced knee. This is particularly true in patients with fixed deformities that do not achieve satisfactory alignment during the manipulation of the leg before cuts are made. Combining these two technologies in TKA allows the optimization of both patient specific component placement and soft tissue driven adjustments to create well-aligned, well-balanced surgical outcomes. Using the presented combination of technologies, the 87% success rate in achieving a quantitively balanced knee through the range of motion is on par with previously reported results on manual surgeries relying on sensor feedback [10,26]. 

Intra-operative load feedback provides guidance for multiple balancing techniques, including the use of robotics to make fine bone cut adjustments. For the latter, the robotics platform presented an additional advantage over manual surgery, as it allowed more detailed and quantified adjustments of the implant component positioning after the initial trialing. This improved the predictability of the bone recuts in relation to their effect on the intra-articular loads. In addition, a femoral recut was more easily facilitated through the 3D planning capabilities of the image-based navigation system built into the robotics platform. Thus, this study illustrates that adjustments after the initial cuts are common and each step taken should consider component alignment, long limb alignment in at least two planes, and soft tissue balancing. The combination of these adjustments generally reduced the intra-articular loads but, more importantly, also avoided outliers. Focusing on the load balancing specifically, the average load was reduced by approximately 7 lbf between the initial cuts and final component positioning. Meanwhile, the standard deviation was drastically reduced while also avoiding conditions with (near) zero and/or excessive intra-articular loads. As such, the use of sensor feedback can be seen as a means of a tighter control on the quality of the balancing step in TKA for the individual patient. This observation holds irrespective of the targeted tibiofemoral loads, with this paper focusing on equal medial-lateral loads; though some recent biomechanical studies point towards a looser lateral side in flexion [27]. While achieving a quantitatively balanced knee was seen as a primary driver during the surgery, a strictly bound coronal alignment was not targeted. This contrasts some of the traditional thinking around mechanical alignment [3] and approaches the functional component alignment recently described by Oussedik et al. [2]. Overall, as shown in Figure 7, the coronal deformity was reduced during surgery for this cohort. Even though the initial cut was made within 2° from mechanical alignment on the tibia, the final coronal alignment, after surgical corrections, ranged between 6° valgus and 9° varus. This reduction in coronal alignment necessitates a significant number of soft-tissue corrections. Indeed, a strong link is observed between the patient’s pre-operative coronal alignment and the need for specific soft tissue corrections. Lateral structures are increasingly addressed as the valgus deformity increases, while the medial structures are increasingly addressed for more severely deformed varus knees. Evaluating the coronal alignment during surgery, our data additionally indicates that the correctable alignment is not uniquely correlated with the final coronal alignment. This is attributed to the change in coronal alignment as a result of the various adjustments that are performed following the initial trialing, as well as the aforementioned soft tissue corrections that are oftentimes indicated. 

An interesting secondary finding of the current study is that fine tuning gaps and joint balance at both extension and flexion predictably provides mid-flexion stability with this particular implant design. This is likely due to the single radius nature of the implant in the sagittal plane throughout mid-flexion. This finding is relevant from a surgical perspective as mid-flexion instability is challenging to correct by adjusting component position or performing soft tissue releases. Nevertheless, care shall be given not to shift the joint line excessively, a situation known to create mid-flexion instability and/or tightness in deep flexion [28]. Using the intra-operative CT-based planning platform, this can be visually verified during surgery and was, as such, likely achieved in the presented series. 

This study has a number of limitations. First, it represents a single surgeon’s case series and, as such, reflects the particular experience of this surgeon with the technologies discussed in this paper. A second limitation links to the strict focus of this paper on the surgical technique in achieving a balanced knee using a robotics platform in combination with an instrumented tibial trial. As such, patient reported outcomes (PROs) have not yet been included in this manuscript, though collection of these PROs is part of an ongoing effort and will be considered for future publications. A third limitation relates to the tibiofemoral load targets that were selected. This paper focuses primarily on equal medial-lateral loads through the range of motion, which have shown clinical benefit in previous controlled trials [7]. However, one can argue from biomechanical analyses that a looser lateral side in flexion, with lower loads, is closer to the intact, healthy knee [27]. In terms of alignment, changing the load targets accordingly could primarily affect the achieved rotation of the femoral component, an aspect that is beyond the scope of this paper.

In conclusion, this study evaluates the surgical technique for a consecutive series of 200 patients receiving a total knee replacement while combining robotics technology with intra-operative sensor feedback. The combination of these technologies allowed achieving a well-balanced knee through the range of motion and avoiding intra-articular load outliers. The initial cuts where guided by a correctable pose in extension and an intra-articular tensor in flexion. Despite the deviation from strict mechanical alignment, soft tissue corrections, bone recuts, and cement adjustments were indicated to achieve a well-balanced, well-aligned knee. This study furthermore indicated that, using this particular implant design and robotics platform and achieving a balanced knee in extension (10°) and flexion (90°) almost consistently resulted in a balanced knee in mid-flexion. 

## Figures and Tables

**Table 1:** Patient demographics in study.

	Age [Year]	BMI [kg/m^2^]	Sex [-]	Pre-Operative Coronal Deformity [°]
Min	48.6	18.8	71.9% Female	24.0 valgus
Mean (Standard Deviation)	70.1 (8.2)	30.4 (5.4)		4.7 varus (9.0)
Max	90.5	47.2		25.0 varus

**Table 2:** Observed intra-articular loads per compartment after initial cuts and following balancing.

	Initial Trialing	After Balancing	Mann–Whitney U-Test
M10 [lbf]	25.0 (12.0)	18.8 (6.4)	*p* < 0.0001
L10 [lbf]	20.5 (11.1)	16.1 (7.0)	*p* = 0.0054
M90 [lbf]	23.0 (12.8)	17.9 (8.2)	*p* = 0.0011
L90 [lbf]	23.1 (11.8)	17.1 (7.2)	*p* < 0.0001

## Data Availability

Restrictions apply to the availability of the data. Data was obtained from Illinois Bone and Joint Institute and are available from the authors with permission of Illinois Bone and Joint Institute.
